# The Lack of Clinical Applications Would be the Cause of Low Interest in an Endothelial Dysfunction Classification

**DOI:** 10.5935/abc.20170019

**Published:** 2017-02

**Authors:** Livia Arcêncio, Paulo R. B. Evora

**Affiliations:** Faculdade de Medicina de Ribeirão Preto - Universidade de São Paulo, São Paulo, SP - Brazil

**Keywords:** Endothelium / dysfunction, Classification, Cardiovascular Diseases, Prevention, Risk Factors

**Figure 1:**
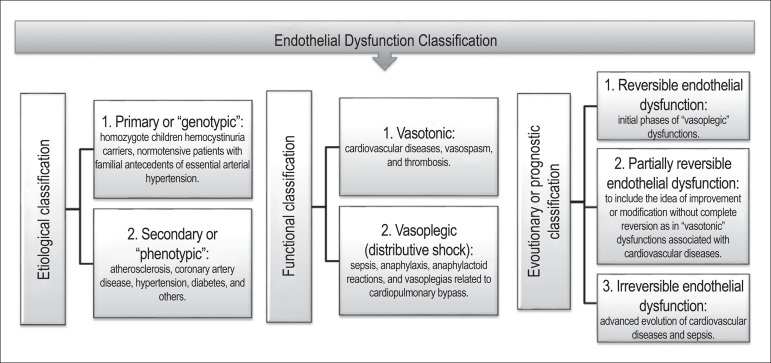
Proposal of an endothelial dysfunction classification. Modified from Evora et al.^1^

Based on the assumption that a classification system is a very critical subject and may significantly improve the prediction of individual responses to treatment and related diseases, we proposed 16 years ago a classification for endothelial dysfunction including etiological, functional, and evolutionary aspects (Figure 1).^1^

Since our first publication, we wrote that a proposition for an endothelial dysfunction classification might deserve criticism because it could still be seen as unsuitable and pretentious. The first question is of a philosophical nature because the present concepts on endothelial function and dysfunction might eventually change dynamically over time. The classification could also be interpreted as a premature reductionism, sounding like an "end of the question" proposal. The lack of clinical applications could be the cause of the low interest in an endothelial dysfunction classification. This editorial aims to explore the differences among the three classification axes and the practical and clinical implications of each proposed category. Aspects relevant to the etiology of the dysfunctions, in addition to treatment directions, are also considered.

The dysfunction in the endothelial cell precedes the organic cellular dysfunction in most cardiovascular diseases and characterizes the primary endothelial dysfunction (etiological classification).^2^ The endothelial dysfunction may be primary (or genetically inherited). This implies a need for the development of diagnostic methods applied to early detection and primary prevention of endothelial dysfunction as a useful measure to halt the development of cardiovascular diseases. Treatment in these cases is aimed at preventing cardiovascular risk factors through lifestyle modifications, such as diet and weight control, physical exercise, and smoking cessation.^3^ From this point of view, endothelial dysfunction should be considered a public health problem. A secondary (or phenotypic) endothelial dysfunction may occur when endothelial cells lose their ability to produce nitric oxide (NO) and increase the expression of vasoconstrictor, proinflammatory, and prothrombotic factors, configuring a proatherosclerotic scenario. Such phenotypic alterations contribute to the formation, progression, and rupture of atherosclerotic lesions, and are commonly found in hypertension, coronary artery disease, and diabetes.^4^ In this type of endothelial dysfunction, pharmacological treatment shows consistent results in terms of restoring the endothelial function. For example, antihypertensive medications to control blood pressure, statin treatment to reduce LDL cholesterol levels, and antidiabetics to reduce blood glucose levels.^5^

Studies in the 1990s definitively established the role of the endothelium in all cardiovascular diseases. Such diseases are associated with endothelial dysfunction due to impaired release of endothelium-derived relaxing factors and, consequently, a risk of spasm and thrombosis (atherosclerotic or nonatherosclerotic obstructive coronary disease, hypertension, diabetes, dyslipidemia, atherosclerosis, Raynaud's phenomenon, and heart failure, among others).^6,7^ Therapeutic interventions have been developed for this type of endothelial dysfunction (vasotonic), which is characterized by functional impairment, aiming to improve the endothelial function and prevent its dysfunction in asymptomatic individuals and in patients with coronary artery disease. Beta-blockers, statins, angiotensin-receptor antagonists, angiotensin-converting enzyme inhibitors, antioxidants, and insulin sensitizers show benefits in these cases. Other substances, such as L-arginine, tetrahydrobiopterin, and folic acid, are also under investigation for their contribution to improving the endothelial function.^8-10^

The vasoplegic endothelial dysfunction classification includes the characteristic situations of severe vasoplegias, many of which are time resistant to the action of vasoconstrictive amines. This type of dysfunction is characterized by an excessive production of vasorelaxant substances produced by the endothelium, especially NO, and include, for instance, vasoplegias during and after cardiopulmonary bypass, sepsis, and anaphylactoid and anaphylactic reactions.^11^ The vasoplegic syndrome has a multifactorial genesis and, in the case of patients undergoing cardiac surgery, occurs mainly due to exposure of the body to nonphysiological materials and the use of heparin/protamine,^12^ triggering an inflammatory response syndrome. During this process, there is complement activation, cytokine release, leukocyte activation, and expression of adhesion molecules, as well as a production of oxygen free radicals, arachidonic acid metabolites, platelet activity factor, NO, and endothelin. The consequences of the inflammatory response syndrome may lead to dysfunction of multiple organs and systems, such as the one that occurs in septic shock. The decrease in systemic vascular resistance observed in vasoplegic syndromes is associated with excessive NO production and may be reversed by NO synthase (NOS) inhibitors and methylene blue.^13^

The term "vasoplegic endothelial dysfunction" was created as part of the proposed classification and deserves some comments. Searching the MEDLINE database using quoted terms, we found: "endothelium dysfunction" (37,640 papers), "endothelial dysfunction" (69,115 papers), "vasoplegic endothelial dysfunction" (12 papers), "vasoplegia" (206 papers), and "vasoplegic syndrome" (243 papers). Assuming that the excessive release of NO is, in fact, an endothelial dysfunction, this terminology would be unified to the search of distributive shock (sepsis, anaphylaxis), anaphylactoid reactions, and vasoplegias related to cardiopulmonary bypass. In this manner, this issue demands special attention from the scientific community, at least in terms of unifying the terminology.^1^

Endothelial dysfunction may be reversible or partially reversible in such cases, according to the prognostic or evolutionary classification. Endothelial dysfunction should be considered in hypertensive postmenopausal women presenting with abnormal endothelium-dependent vascular function. However, a significant improvement in endothelial function may be reached after 6 months of antihypertensive therapy. These changes may identify patients with a more favorable prognosis.^14^ Dysfunction of the coronary or peripheral vascular endothelium is an independent predictor of cardiovascular events and provides valuable prognostic information. In such cases, modification of risk factors and drug treatment (statins and angiotensin-converting enzyme inhibitors) may improve the endothelial function and prognosis.^15^ Most risk factors related to atherosclerosis and cardiovascular morbidity and mortality have been found to be associated with the endothelium.^14^ These risk factors include hyperlipidemia, hypertension, diabetes, and smoking, which may be reversed by pharmacological or nonpharmacological treatment. In other words, it is possible to improve endothelial dysfunction using medical treatment and exercise, even without completely reversing it.^16,17^

Irreversible endothelial dysfunction usually occurs during the progression of cardiovascular diseases and sepsis.

We have been using the proposed classification since 2000^1^ as a didactic model, carefully emphasizing eventual biases concerning its misinterpretation. However, the current usefulness of an endothelial dysfunction classification still remains "an open discussion". Semiquantitative measurements of endothelial dysfunction may potentially amend the assessment of the proposed categories. We hoped that the classification system would be used to improve and uniformly diagnose patients, in addition to providing a route for collaborative studies on endothelial dysfunction across academic centers. However, as already mentioned, the lack of clinical applications could be the cause for the low interest in an endothelial dysfunction classification. Perhaps the development of biomarkers may strengthen the clinical reasoning of cardiovascular diseases from the point of view of endothelial dysfunction.^17-19^
